# Lentils and Gluten Cross Contact

**DOI:** 10.3389/fnut.2022.867954

**Published:** 2022-04-29

**Authors:** Tricia Thompson, Trisha Bury Lyons, Amy Keller

**Affiliations:** ^1^Gluten Free Watchdog, LLC, Manchester-by-the-Sea, MA, United States; ^2^Department of Clinical Nutrition, MetroHealth Medical Center, Cleveland, OH, United States; ^3^Mary Rutan Hospital, Bellefontaine, OH, United States

**Keywords:** lentils, gluten, cross contact, celiac disease, FDA recalls

## Abstract

Lentils are naturally gluten-free and are recommended for people with celiac disease and other gluten-related disorders. However, like oats, they appear to be at a heightened risk of cross contact with gluten-containing grains. The purpose of this study was to spot check for the presence of errant gluten-containing grains in a variety of brands of lentils purchased in 2021. Twenty-five bags of different dry lentil products representing 24 brands were purchased online and at various grocery stores. Each bag of lentils was individually hand sorted. Two of the 25 packages of lentils contained errant gluten-containing grains. One 16-ounce (454 g) bag contained a grain of wheat. Another 16-ounce (454 g) bag contained a grain of wheat and a grain of barley. For a product to be considered gluten-free in the United States, it must contain <20 mg of gluten per kilogram (or 20 parts per million of gluten). A product at the 20-ppm level of gluten should contain no more than 2 intact gluten-containing grains per kilogram or 35.27 ounces (1,000 g) of food (or 1 intact gluten-containing grain in 17.64 ounces [500 g] of food). Based on these calculations, a 16-ounce (454 g) bag of lentils containing 1 intact gluten-containing grain would not be considered gluten-free. Lentils are at risk of cross contact with gluten-containing grain. Consumers should continue to sort through lentils removing foreign grain, and rinse sorted lentils under running water to remove grain dust before cooking.

## Introduction

Lentils are naturally gluten-free. They are recommended for people with celiac disease and other gluten-related disorders. However, like oats, they appear to be at a heightened risk of cross contact with gluten-containing grains. This is due in part to farming practices. Lentils may come into contact with wheat, barley, or rye while being grown, harvested, and/or stored. The United States Department of Agriculture, Agricultural Marketing Service, Federal Grain Inspection Services standard for lentils allows for a certain percentage of foreign material (i.e., all matter other than lentils) (1). Acceptable percentages range from 0.2 to 0.5% foreign material depending on the grade of dockage-free lentils.

Between March of 2016 and April of 2020, Gluten Free Watchdog (GFWD) received at least 17 complaints from consumers about errant grain in dry lentils. These complaints represented 9 different brands. All but one product were labeled gluten-free or certified gluten-free. In the US, all products under the labeling jurisdiction of the Food and Drug Administration (FDA) and labeled gluten-free, must comply with the FDA's gluten-free labeling rule, including containing a level of gluten below 20 parts per million (ppm). There are multiple organizations in the US certifying gluten-free foods. All have differing criteria for certification, including containing a level of gluten from below 5 ppm up to <20 ppm. Complaints submitted by consumers generally included photographs of the foreign material. In most cases, GFWD was able to determine that the errant grain included wheat and/or barley either through laboratory testing or by expert review of the grain photographs by farmers familiar with gluten-containing grain.

GFWD also sorted an unopened bag of a brand of lentils reported by a consumer. It contained numerous errant grains. It was determined through laboratory testing that the grain found included gluten-containing grain (e.g., results well above 20 ppm of gluten). As a result of consumer complaints and testing commissioned by GFWD, GFWD has been advising consumers with celiac disease or another gluten-related disorder to carefully sort dry lentils removing any foreign grain and to rinse sorted lentils under running water to remove grain dust.

**Figure 1:**
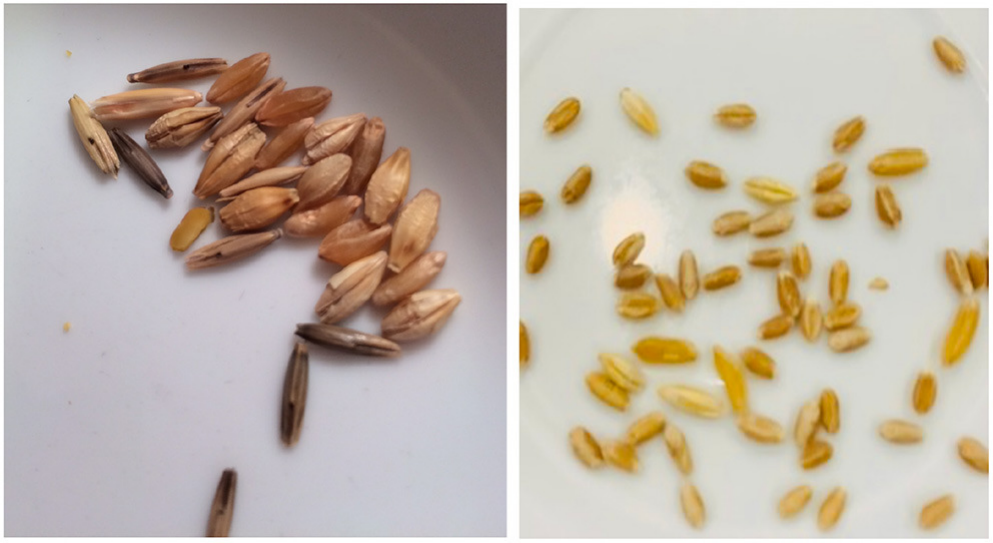
Errant grain found in two different brands of labeled gluten-free lentils later recalled by FDA. Photo credit (left) Kathleen Warthen.

Of the 8 products labeled gluten-free or certified gluten-free reported by consumers that contained errant grain, GFWD reported 6 to the FDA. Consumers were advised to report the other 2 products to an FDA consumer complaint coordinator. The 2 products with the most egregious misbranding in terms of the number of errant grain found, were recalled (2, 3) (Figure 1). To the best of the investigators' knowledge, no public enforcement action was taken against the manufacturers of the other 6 reported products.

The purpose of this study was to spot check for the presence of errant gluten-containing grains in a variety of brands of lentils purchased in 2021, and to assess whether there had been any changes to gluten-free claims on product packaging of the lentils originally reported by consumers to GFWD.

## Methods

During the first half of 2021, 25 bags of different dry lentil products representing 24 brands were purchased online through Amazon, Wal-Mart, and Vitacost, and at various grocery stores in Ohio, US. Products had best by dates ranging from July 2021 to February 2025. Products varied in weight from 10 ounces (283 g) to 35 ounces (992 g). Each bag of lentils was individually hand sorted. Lentils were scooped from product packaging in 2-tablespoon increments and poured onto a flat white tray. They were separated and sorted with a paring-style knife. This process was repeated until each bag of lentils was empty. All foreign material was set aside for later review. Seven of the 9 brands of lentils previously reported by consumers to GFWD were included in the sorting. Two brands could not be included due to the lack of availability. Product labels were also reviewed for gluten-free claims and other advisory statements.

## Results

### 2021 Lentil Sorting and Label Review

**Figure 2:**
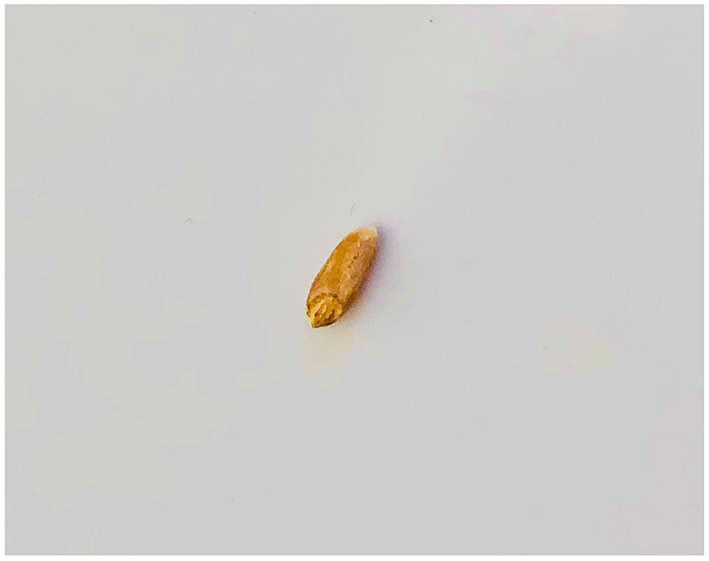
Errant wheat grain.

**Figure 3:**
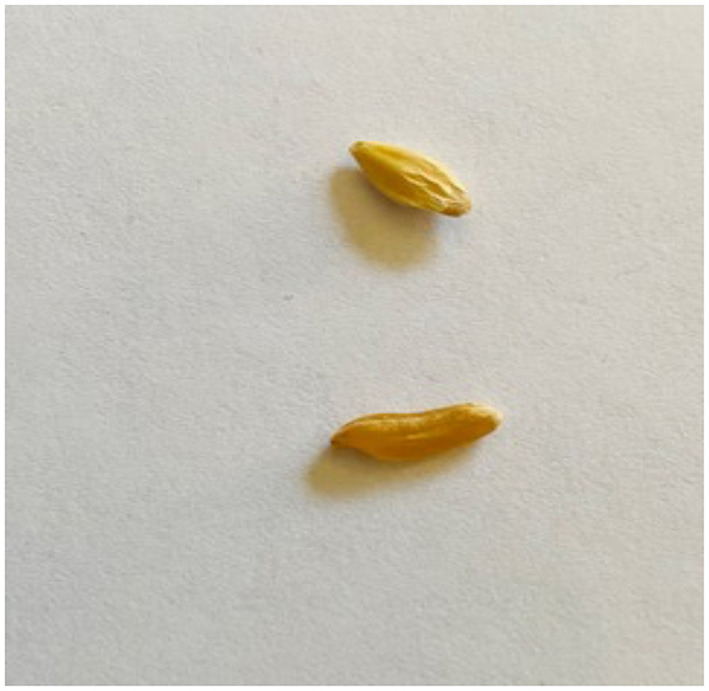
Errant wheat and barley grain.

Two of the 25 packages of lentils (8%) contained errant gluten grains (Table 1). One of these 16-ounce (454 g) bags contained a grain of wheat (Figure 2). The other 16-ounce bag (454 g) contained a grain of wheat and a grain of barley (Figure 3). While neither of these products was labeled gluten-free, it is important to note that a 16-ounce (454 g) bag of lentils containing 1 gluten-containing grain would not be considered gluten-free. Packaging for both products included an allergen advisory statement for wheat (e.g., may contain traces of wheat, may contain wheat). Non-gluten-containing foreign material was found in 6 additional bags of lentils. This material included split peas, mallow seeds, stones, and rice grain.

**Table 1:** Errant gluten-containing grain findings and labeling language on packages of lentils hand sorted in 2021.

**#**	**Weight (oz)**	**Ingredients**	**Allergy advisory statement for wheat?**	**Other advisory language?**	**Labeled GF?**	**Errant gluten grains found?**
1	16	Organic green lentils	No	Under cooking instructions: “Before cooking always examine, sort, and rinse well to ensure maximum quality of this natural product.”	No	No
2	12	Organic red lentils, organic green lentils, and organic black lentils	No	“Lentils are an organic agricultural product. Despite the use of modern cleaning equipment it is not always possible to remove all foreign material. Always rinse and sort lentils before cooking.”	No	No
3	27	Lentils	No	“Beans are natural agricultural products. Despite use of modern agricultural equipment, it is not always possible to remove all foreign material. Sort and rinse before cooking.”	No	No
4	16	Organic French green lentils	Packaged in a facility that also processes tree nuts and wheat.	No	No	No
5	16	Not listed	No	“Dried beans are naturally grown, raw agricultural products. Although they have been mechanically cleaned before packaging, some foreign material may be present. Wash and carefully sort before cooking.”	No	No
6	15.5	Lentils	Facility is not dedicated gluten-free.	“Dry beans/peas/lentils are a natural agricultural product. Despite use of modern cleaning equipment, foreign material and grains may be present. Please inspect, sort, and rinse product before cooking.”	Yes	No
7	16	Lentils	No	“Lentils are a natural agricultural product. Despite the use of modern processing equipment, it is not always possible to remove all foreign material. Before cooking, sort lentils to remove small stones or soil. Rinse under running water.”	No	No
8	35	Organic brown lentils	No	No	Yes, certified	No
9	32	Hulled chickpea, mung bean, hulled lentils, hulled pigeon pea, black gram	No	No	Yes	No
10	16	Green lentils (gluten free)	No	No	Yes	No
11	16	Lentils	Facility is not dedicated gluten-free.	“Dry beans/peas/lentils are a natural agricultural product. Despite use of modern cleaning equipment, foreign material, and grains may be present. Please inspect, sort, and rinse product before cooking.”	Yes	No
12	16	Lentils	No	“Dried beans are an agricultural product and may contain foreign material. Sort and wash prior to cooking. Dry beans must be cooked prior to consumption.”	Yes	No
13	16	Lentils	May contain traces of wheat due to crop rotation.	“Lentils are a natural agricultural product. Despite use of modern cleaning equipment, it is not always possible to remove all foreign material. Sort and rinse lentils before cooking.”	No	No
14	16	Lentils	No	“Beans are a natural agricultural product. Despite use of modern cleaning equipment, it is not always possible to remove all foreign material. Carefully sort and rinse before cooking.”	No	No
15	16	Lentils	May contain soybean and wheat.	“Beans are harvested in their natural state. Even with the most careful attention and best processing equipment available, we suggest you look over the contents closely to pick out any foreign substances such as small stones, soil particles, etc. As a helpful hint, rinse the beans with drinkable water to provide a wholesome dish.”	No	No
16	16	Lentils	May contain traces of wheat.	“Lentils are a natural agricultural product. Despite use of modern cleaning equipment, it is not always possible to remove all foreign material. Sort and rinse lentils before cooking.”	No	Yes (wheat)
17	16	Organic green lentils	No	“Cooking Instructions: Rinse and sort lentils.”	Yes	No
18	10	Organic sprouted green lentils	No	No	No	No
19	16	Organic green lentils	May contain wheat.	“Before cooking, always examine, sort and rinse well to assure maximum wholesomeness of this natural product.” “Lentils are an agricultural product. Despite use of modern cleaning equipment, it is not always possible to remove all foreign material. Sort and rinse lentils before cooking.”	No	Yes (wheat) (barley)
20	16	Organic green lentils	Packaged on equipment that handles wheat.	No	Yes	No
21	15	Organic brown lentils	This product was packaged in a facility that also handles wheat, soy, and tree nuts.	“May contain agricultural debris. Sort before cooking.” First sentence in the directions is “sort & rinse.”	No	No
22	16	Lentils	May contain traces of soy and wheat.	No	No	No
23	16	Organic French green lentils	No	“Despite use of modern cleaning equipment, it is not always possible to remove all foreign material. Sort and remove any debris. Rinse under running water.”	Yes	No
24	16	Red lentils	May contain wheat.	“Carefully sort lentils, removing any debris or shriveled lentils, and rinse thoroughly.”	No	No
25	16	Whole green lentils	May contain traces of wheat.	“Carefully sort and rinse lentils before cooking.”	No	No
Totals:			12/25 product labels included an allergen advisory statement for wheat/gluten	18/25 product labels included other advisory language that includes sorting and rinsing lentils	9/25 products labeled GF, including 1 certified GF	2/25 products contained errant gluten grains

Nine of the 25 products (36%) were labeled gluten-free. Of these, 1 was labeled certified gluten-free. Twelve of the 25 products (48%) contained an allergen advisory statement for wheat or gluten, including 3 products labeled gluten-free. Eighteen of 25 products (72%) contained a statement advising consumers to sort and rinse lentils, including 5 products labeled gluten-free.

### Changes to Gluten-Free Labeling Claims Over Time

Of the 9 brands of lentils reported to GFWD by consumers, 8 were labeled gluten-free, including 3 that carried a certified gluten-free mark on product packaging at the time the complaint was received. Six of the 8 brands originally labeled or certified gluten-free were reviewed in 2021. Two could not be reviewed because they were not available for retail purchase. Of the brands that could be compared, 3 of the 6 original consumer-reported products were no longer labeled gluten-free in 2021 (Table 2). Three of the original consumer-reported products included a certified gluten-free mark on product packaging. None of these 3 products were certified gluten-free in 2021. Two of these products went from labeled certified gluten-free to labeled gluten-free. One product went from labeled certified gluten-free to no gluten-free claim. This product was 1 of 2 lentil products that underwent a recall. None of the 6 products reviewed in 2021 were found to contain errant grain.

**Table 2:** Errant gluten-containing grain findings and labeling language changes in 2021 as compared to dry lentil brands reported by consumers between 2016 and 2020.

**Consumer (C) Researcher (R)**	**#**	**Labeled GF?**	**Errant gluten grains found?**	**Reported to FDA by GFWD?**
C	2	Yes, certified	Yes	Yes; recalled
R	2	No	No	Not applicable
C	6	Yes	Yes	Yes
R	6	Yes	No	Not applicable
C	10	Yes, certified	Yes	Consumer advised to file a complaint with FDA
R	10	Yes	No	Not applicable
C/R^*^	22	Yes	Not applicable^*^	Yes
R	22	No	No	Not applicable
C	23	Yes, both certified and not certified	Yes	Yes
R	23	Yes	No	Not applicable
C	24	Yes	Yes	Yes
R	24	No	No	Not applicable
C	25	No	Yes	No; product not labeled gluten-free
R	25	No	No	Not applicable
C	26	Yes	Yes	Yes, recalled
R	26	Not applicable	Not applicable	Not applicable
C	27	Yes	Yes	Consumer advised to file a complaint with FDA
R	27	Not applicable	Not applicable	Not applicable

* *General consumer complaints received for this brand of lentils. Gluten Free Watchdog tested the product for gluten*.

*Some results were >20 parts per million of gluten*.

## Discussion

Two, 16-ounce (454 g) bags sorted in 2021 were found to contain errant barley and/or wheat. One of the bags contained a single grain and the other bag contained 2 grains. According to calculations published by the Gluten-Free Certification Organization, one gluten-containing grain could contain upwards of 10.5 mg of protein, including 9.45 mg of gluten (4). According to the study authors, the protein and gluten levels are based on the upper limit of published ranges (i.e., a single grain weight of 50 mg, 21% grain protein, and a gluten content as 90% of the protein content of the grain) (4). For a product to be considered gluten-free in the United States, it must contain <20 mg of gluten per kilogram (or 20 parts per million of gluten). A product at the 20-ppm level of gluten should contain no more than 2 intact gluten-containing grains per kilogram or 35.27 (1,000 g) ounces of food (or 1 intact gluten containing grain in 17.64 ounces [500 g] of food) (4, 5). Based on these calculations, a 16-ounce (454 g) bag of lentils containing 1 intact gluten-containing grain would not be considered gluten-free.

Ideally, the gluten level in lentil samples would be determined using a sandwich R5 ELISA. However, testing is problematic when sample extractions are not representative of the sample as a whole. Trying to homogenize or evenly distribute one or two grains of wheat or barley within a 16-ounce (454 g) sample of whole lentils is challenging, and similar to trying to evenly distribute a grain of wheat or barley within a sample of intact inherently gluten-free grains (4). Thus, hand sorting of lentils for errant gluten-containing grain is a viable method for determining gluten levels. However, it is possible that grain dust was present in the samples that would not be accounted for via hand sorting.

It is important to consider the safety of dry lentils on a per serving basis. The threshold daily intake of gluten that is considered by experts to be tolerable for most people with celiac disease is 10 mg (6). If a single errant gluten-containing grain ends up in a serving of cooked dry lentils, the consumption of this single grain is almost enough to cause the 10 mg threshold to be reached.

Lentils are a naturally gluten-free food, yet only 36% of the lentil products sorted for this project in 2021 included a gluten-free claim on product packaging. Among the brands of lentils originally reported to GFWD by consumers, there appears to be a trend toward removing the gluten-free claim from product packaging or no longer certifying the lentils as gluten-free. Of those lentils that were labeled gluten-free, one-third or 3 included an allergen advisory statement for wheat. Allergen advisory statements such as “packaged on equipment that handles wheat” or “facility is not dedicated gluten-free” are very confusing to consumers, especially when these statements are made alongside gluten-free claims. While it is impossible to know the reason for these labeling changes, it is plausible that the FDA recalls led to increased industry awareness of gluten cross contact issues in lentils. Regardless of the presence or absence of a gluten-free labeling claim, this increased awareness may have resulted in cleaner lentils.

It is important for consumers to understand that in the US, allergen advisory statements for wheat are allowed on the labels of foods making gluten-free claims. Regardless of their presence, foods labeled gluten-free must be in compliance with the gluten-free labeling rule. Allergen advisory statements are voluntary and not covered under any federal regulation. Research suggests that the presence or absence of an allergen advisory statements for wheat is not a useful indicator of the gluten-free status of a food.” (7, 8).

Lentils may be particularly susceptible to cross contact with wheat and barley due to crop rotation or proximity to grain fields, the use of shared harvesting equipment, and/or the use of shared storage facilities (e.g., grain silos) (5). Lentils grow in cooler climates similar to wheat and barley (9, 10). As a result they are frequently grown in rotation with or in fields adjacent to these grains. In a crop rotation, lentils are grown after wheat or barley because these grains are less likely to pass along diseases harmful to lentils (9, 10). While the above is true for other crops such as chickpeas and dry peas (9, 10), size and color differences between these crops and barley and wheat grain may make sorting somewhat easier.

None of the above is meant to suggest that those with celiac disease or other gluten-related disorders should not eat lentils. For the present study, 27 pounds of lentils were sorted. Only 3 errant gluten-containing grains were found. Given the farming practices for lentils, this suggests fairly robust sorting and cleaning before the lentils are packaged for the consumer. Regardless, the gluten-free consumer should continue hand-sorting lentils, removing any foreign material, and rinsing under running water using a fine-mesh colander to remove any grain dust. When available, consumers may want to choose lentils that are labeled gluten-free. Lentils grown under a gluten-free purity protocol, following steps similar to what is followed for gluten-free purity protocol oats (i.e., protocols to control the presence of gluten-containing grain throughout the entire process of growing, harvesting, milling, and processing) (11) are a new option for consumers (12).

Manufacturers of gluten-free foods may use whole lentils or lentil flour in their products. Results of a search on the terms “gluten-free” and “lentil” on a popular mail order site in the US, included chips, crackers, pasta, ready-to-eat breakfast cereal, soup, and rice mixes. It would be prudent of manufacturers to ask suppliers for certificates of analysis for gluten for lentil ingredients. In addition, manufacturers should consider sorting whole lentils for gluten-containing grain and testing lentil flour for gluten.

## Conclusion

Lentils are at risk of cross contact with gluten-containing grain due to various farming practices. While there appears to be movement away from labeling lentils gluten-free, the vast majority of lentil products sorted in 2021 were free of wheat, barley, and rye grain. Regardless, due to the continued risk of cross contact, consumers should sort through and rinse lentils before cooking. When available, lentils labeled gluten-free should be chosen over products not labeled gluten-free. Consumers concerned about cross contact also may want to choose lentils grown and processed following a gluten-free purity protocol.

## Data Availability Statement

The original contributions presented in the study are included in the article/supplementary material, further inquiries can be directed to the corresponding author/s.

## Author Contributions

All authors contributed to the study design, the interpretation of the data, drafting of the manuscript, and approve the submitted version.

## Funding

Gluten Free Watchdog, LLC, paid for the cost of the lentils.

## Conflict of Interest

TT is the owner and founder of Gluten Free Watchdog, LLC. The remaining authors declare that the research was conducted in the absence of any commercial or financial relationships that could be construed as a potential conflict of interest.

## Publisher's Note

All claims expressed in this article are solely those of the authors and do not necessarily represent those of their affiliated organizations, or those of the publisher, the editors and the reviewers. Any product that may be evaluated in this article, or claim that may be made by its manufacturer, is not guaranteed or endorsed by the publisher.
